# Role of Carbohydrate response element-binding protein in mediating dexamethasone-induced glucose transporter 5 expression in Caco-2BBE cells and during the developmental phase in mice

**DOI:** 10.1080/19768354.2023.2301009

**Published:** 2024-01-04

**Authors:** Soonjae Hwang, Sangbin Park, Jaewan Kim, Ah-Reum Oh, Ho-Jae Lee, Ji-Young Cha

**Affiliations:** a Department of Biochemistry, Lee Gil Ya Cancer and Diabetes Institute, College of Medicine, Gachon University, Incheon, Republic of Korea; b Department of Health Sciences and Technology, GAIHST, Gachon University, Incheon, Republic of Korea

**Keywords:** Fructose transporter, glucocorticoid hormone, dexamethasone, ChREBP, small intestine

## Abstract

Glucose transporter 5 (GLUT5), the main fructose transporter in mammals, is primarily responsible for absorbing dietary fructose in the small intestine. The expression of this intestinal gene significantly increases in response to developmental and dietary cues that reach the glucocorticoid receptor and carbohydrate response element-binding protein (ChREBP), respectively. Our study demonstrates that ChREBP is involved in the dexamethasone (Dex)-induced expression of *GLUT5* in Caco-2BBE cells and the small intestine of both wild-type and ChREBP-knockout mice. Dex, a glucocorticoid, demonstrated an increase in *GLUT5* mRNA levels in a dose- and time-dependent manner. While the overexpression of ChREBP moderately increased *GLUT5* expression, its synergistic increase in the presence of Dex was noteworthy, whereas the suppression of ChREBP significantly reduced Dex-induced *GLUT5* expression. Dex did not increase ChREBP protein levels but facilitated its nuclear translocation, thereby increasing the activity of the *GLUT5* promoter. *In vivo* experiments conducted on 14-day-old mice pups treated with Dex for three days revealed that only wild-type mice (not ChREBP-knockout mice) exhibited Dex-mediated *Glut5* gene induction, which further supports the role of ChREBP in regulating GLUT5 expression. Collectively, our results provide insights into the molecular mechanisms involved in the regulation of GLUT5 expression in response to developmental and dietary signals mediated by glucocorticoids and ChREBP.

**General significance:** The transcription factor ChREBP is important for Dex-mediated *Glut5* gene expression in the small intestine.

## Introduction

Glucose transporter 5 (GLUT5), which is essential for fructose absorption in the small intestine (Douard and Ferraris 2008; Barone et al. 2009; Kim et al. 2017; Oh et al. 2018), undergoes notable changes in expression during developmental transitions, particularly from the suckling to the weaning phase in mammals (Wijtten et al. 2011; Navis et al. 2019; Beaumont et al. 2020). This process is important as it dictates the capacity of the intestine to absorb dietary fructose during the weaning phase (Shu et al. 1997), when dietary sources shift significantly (Godbole et al. 1981; Ferre et al. 1986; Beaumont et al. 2020). GLUT5 remains largely unresponsive to fructose during the suckling phase (Douard et al. 2008; Liu et al. 2020), indicating that a tightly regulated mechanism triggers its expression and activity upon weaning.

Glucocorticoids (GCs) are potent metabolic and inflammatory regulators that modulate GLUT5 expression via the glucocorticoid receptor (Magomedova and Cummins 2016; Ramamoorthy and Cidlowski 2016). During the weaning phase, a critical developmental juncture characterized by a shift from a high-fat diet in breast milk to a carbohydrate-rich solid diet (Godbole et al. 1981; Ferre et al. 1986), serum GC levels increase (Blake and Henning 1983; Back et al. 1985; Frank 1991; Yu et al. 2019), which is concomitant with an increase in GLUT5 expression (Douard et al. 2008). Previous studies have demonstrated that dexamethasone (Dex), a synthetic GC, elevates *GLUT5* gene expression in Caco-2 cells by activating the glucocorticoid receptor and binding to the glucocorticoid response element within the *GLUT5* promoter (Mochizuki et al. 2008; Takabe et al. 2008; Inamochi et al. 2016).

In concert with GCs, the carbohydrate response element-binding protein (ChREBP), a transcription factor activated by glucose (Kawaguchi et al. 2001; Witte et al. 2015), has been identified as a pivotal regulator of GLUT5 (Oh et al. 2018). Upon activation, ChREBP translocates to the nucleus, interacts with max-like protein X (Mlx) (Stoeckman et al. 2004; Ma et al. 2006), and binds to the carbohydrate response elements of target genes, including *Glut5* (Oh et al. 2018; Noblet et al. 2021). Previous studies have elucidated the role of ChREBP in fructose-induced GLUT5 expression and demonstrated a substantial decrease in intestinal *Glut5* gene expression in ChREBP-knockout (KO) mice (Oh et al. 2018).

However, no study has investigated whether GC stimulates *Glut5* expression in the absence of ChREBP during the suckling-to-weaning transition, a period that induces intestinal maturation and development. In this study, we investigated the potential function of ChREBP in Dex-induced GLUT5 expression in Caco-2BBE cells and explored the functional role of ChREBP in *Glut5* gene expression in the small intestine of mice during the suckling-to-weaning transition, using ChREBP-KO mice. Our findings aim to shed light on the mechanistic interplay between Dex and ChREBP in modulating intestinal *Glut5* gene expression, providing insights that improve our understanding of the metabolic consequences of altered fructose absorption and utilization within distinct dietary and developmental contexts.

## Material and methods

### Cell culture

The Caco-2BBE cell line (human intestinal epithelial cells) was cultured in 25 mM glucose Dulbecco’s modified Eagle’s medium (DMEM; Welgene, Gyeongsan, Republic of Korea) supplemented with 10% fetal bovine serum (Welgene, Gyeongsan, Republic of Korea) and 1% penicillin–streptomycin at 37°C in a humidified atmosphere containing 5% CO_2_. After 1 d at approximately 70% confluence, Dex, a glucocorticoid receptor agonist (D4902; Sigma-Aldrich, St. Louis, MO, USA), was added to the culture media of the Caco-2BBE cells. Dex was dissolved in absolute ethanol and stored until further use. Unless otherwise stated, all culture media and reagents were purchased from GIBCO Life Technologies (Rockville, MD, USA).

### Mice and Dex injection

Wild-type (WT) C57BL/6J and ChREBP-KO mice were purchased from Jackson Laboratory (Bar Harbor, ME, USA). The mice were kept on a standard diet containing 13% kcal fat, 25% protein, 55% starch, 5% sucrose, and 2% lactose (PicoLab Rodent Diet 20; Orient Bio, Gyeonggi Province, Korea) under a 12-h light/dark cycle. Mouse pups were suckled daily from birth to postnatal day (PND) 21. On PND 14, mice were intraperitoneally injected with Dex (1 mg/kg) for 3 days. Following the last Dex treatment, the pups were isolated from breeding pairs and subjected to a 5-h fast before being euthanized using isoflurane. The duodenum of the small intestine was removed and snap-frozen in liquid nitrogen. All animal studies were performed in accordance with protocols approved by the Institutional Animal Care and Use Committee of the Lee Gil Ya Cancer and Diabetes Institute, Gachon University (LCDI-2021-0114).

### Quantitative PCR (qPCR)

Total RNA was isolated from Caco-2BBE cells and the duodenum of the small intestine using RNAiso Plus (Takara, Shiga, Japan), according to the manufacturer’s instructions. The isolated RNA was reverse transcribed using a high-capacity cDNA synthesis kit (Takara, Shiga, Japan). Gene-specific primers were used for PCR with SYBR® Premix EX Taq™II, ROX Plus (Takara, Shiga, Japan). Results were expressed as 2^-ΔΔCt^, and fold change was determined by comparison to the untreated control group. The primers used for qPCR are listed in Table 1.

**Table 1.** Primer sequences for quantitative real-time PCR.

Species	Gene symbol	Primer sequence (5′ to 3′)	Primer sequence (5′ to 3′)
Human	*GLUT5*	GGCGCTGCAGAACACCAT	AAGGCGTGTCCTATGACGTAGAC
Human	*ChREBP*	GTCTGCAGGCTCGGAACAG	AAGGAGGAAATCAGAACTCAGGAA
Human	*CYCHLOPHYLIN*	TGCCATCGCCAAGGAGTAG	TGCACAGACGGTCACTCAAA
Mouse	*Glut5*	GGCGCTGCAGAACACCAT	AAGGCGTGTCCTATGACGTAGAC
Mouse	*Chrebp*	AGAACCGACGTATCACACACATCT	CAGGGTGTCGAATCCTAGCTTAA
Mouse	*Cyclophilin*	TGGAGAGCACCAAGACAGACA	TGCCGGAGTCGACAATGAT
	* *		

### RNA interference

The small interfering RNA (siRNA) duplex oligonucleotides targeting human ChREBP were selected and synthesized by Invitrogen (StealthTM RNAi, Carlsbad, CA, USA). The control siRNA oligonucleotides were purchased from Genolution (Seoul, Republic of Korea). The siRNA sequences were as follows: siChREBP forward 5′-CCAAGUGGAAGAAUUUCAAAGGCCU-3′, reverse 5′-AGGCCUUUGAAAUUCUUCCACUUGG-3′; siC forward 5′-CCUCGUGCCGUUCCAUCAGGUAGUU-3′; reverse 5′-CUACCUGAUGGAACGGCACGAGGUU-3′. For gene silencing or overexpression, Caco-2BBE cells were transfected with siRNAs or plasmids containing human ChREBP, Mlx, and β-galactosidase. The proximal region of *Glut*5 (–2165/+0) was amplified from mouse genomic DNA using PCR and inserted into the pGL4 basic vector (mGlut5-2165) [8]. All transfections were performed using reverse transfection. RNAiMAX (Thermo Fisher Carlsbad, CA, USA) and X-tremeGENE HP DNA transfection (Roche Diagnostics, Indianapolis, IN, USA) reagents were used for gene silencing and overexpression, respectively. Following 24 h of transfection, fresh DMEM containing 10% fetal bovine serum with 1% non-essential amino acids was added into individual wells of 24-well plates, followed by Dex treatment 24 h after media addition.

### Western blotting

Caco-2BBE cells were washed with sterile phosphate-buffered saline and treated with RIPA buffer containing proteinase inhibitors (Sigma-Aldrich, St. Louis, MO, USA). Cell lysates were prepared and subjected to western blotting as previously described (Moon et al. 2022; Um et al. 2023) using antibodies against ChREBP (NB400-13; Novus Biologicals, Centennial, CO, USA), GLUT5 (27571-1-AP; Proteintech, Rosemont, IL, USA), GAPDH (MAB374; Millipore, St. Louis, MO, USA), and TATA-binding protein (TBP) (ab63766; Abcam, Cambridge, UK). To detect nuclear ChREBP, nuclear lysates of Caco-2BBE cells were obtained using the NE-PER™ Nuclear and Cytoplasmic Extraction Reagents Kit (78833; Thermo Fisher Scientific, Carlsbad, CA, USA), according to the manufacturer’s instructions. Immunoreactive proteins were detected using an ECL kit (Thermo Fisher Scientific, Carlsbad, CA, USA), and the images were processed using Adobe Photoshop (Adobe, San Jose, CA, USA). Immunoreactive proteins were visualized using Amersham ImageQuant 800 (Marlborough, MA, USA). Protein bands were quantified using ImageJ software (National Institutes of Health, Bethesda, MD, USA).

### Luciferase assay

Luciferase reporter plasmids containing the mouse *Glut5* promoter were transfected into Caco-2BBE cells with siRNAs targeting ChREBP or ChREBP/Mlx overexpression vectors. Luciferase activity was determined using the Luciferase Assay System (E1501; Promega Corporation, Fitchburg, WI, USA), according to the manufacturer’s instructions, and the results were expressed as arbitrary units normalized to β-galactosidase activity. All experiments were performed in triplicates and repeated at least three times.

### Statistical analysis

All statistical analyses were performed using the Mann–Whitney U test (GraphPad Prism 8). Statistical significance was set at *p* < 0.05. The error bars represent the standard error of the mean (SEM).

## Results

### Dex-induced *GLUT5* expression is affected by the presence or absence of ChREBP in Caco-2BBE cells

**Figure 1.**
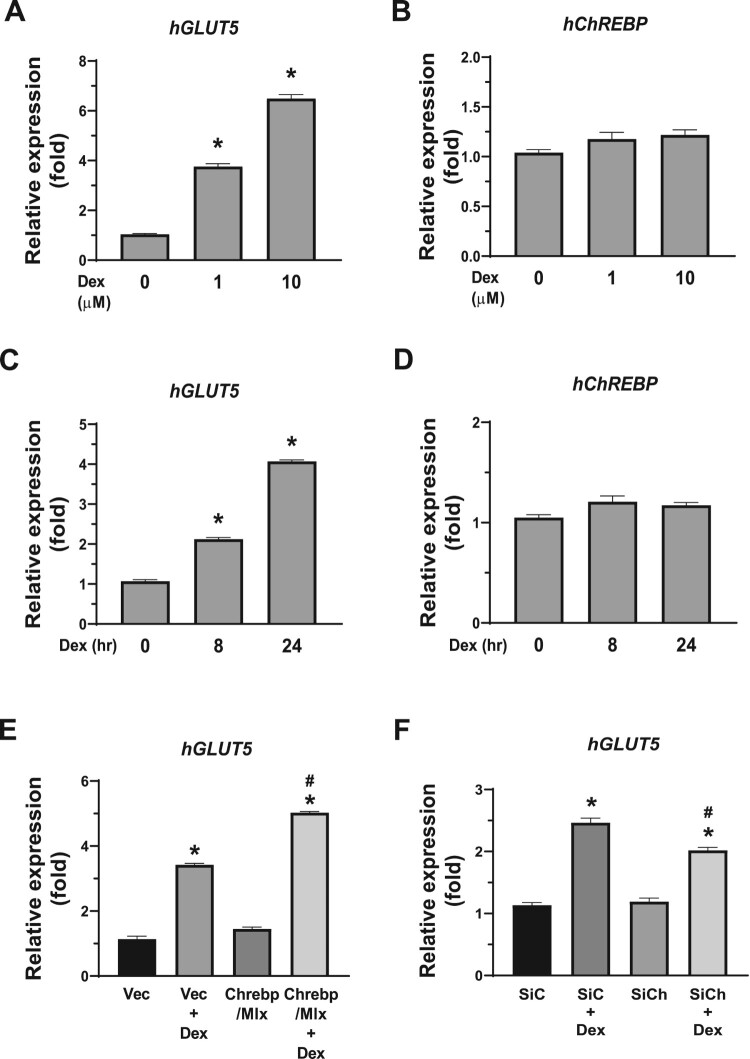
Dexamethasone upregulated *Glut5* gene expression in Caco-2BBE cells via ChREBP. Caco-2BBE cells were treated with dexamethasone (Dex) at the indicated doses or times. (A) The relative mRNA expression of human *GLUT5* (*hGLUT5*) and *ChREBP* was determined using qPCR in Caco-2BBE cells treated with Dex (0 to 10 µM) for 24 h. (B) The relative mRNA expression of *hGLUT5* and *ChREBP* was determined using qPCR in Caco-2BBE cells treated with 1 μM Dex for 0, 8, and 24 h. *p < 0.05. Caco-2BBE cells were transfected with pcDNA-hChREBP/pcDNA-hMlx (C) or siChREBP (D) for 48 h and treated with 1 μM Dex for another 24 h. mRNA expression of *GLUT5* was determined using qPCR. The target gene expression was normalized to the expression of cyclophilin and expressed as mean ± SEM. *p < 0.05 vs. transfection-matched, control group; ^#^*p* < 0.05 vs. Dex-matched, transfection group.

To evaluate the effects of glucocorticoid signaling on the expression of *GLUT*5 and *ChREBP* in intestinal epithelial cells, Caco-2BBE cells were treated with 1 to 10 μM Dex for 24 h or 1 μM Dex for 8 to 24 h (Figure 1). *GLUT*5 mRNA levels increased significantly in a dose-dependent manner, while *ChREBP* mRNA levels remained unaffected by Dex treatment (Figure 1A). Time-dependent gene induction by Dex was observed only for *GLUT*5, not for *ChREBP* (Figure 1B). Caco-2BBE cells were transfected with ChREBP and its heterodimer partner Mlx expression vectors or siRNA to determine whether ChREBP expression levels influenced Dex-induced *GLUT*5 expression. The overexpression of ChREBP/Mlx slightly increased *GLUT*5 expression, which was further synergistically increased by Dex treatment (Figure 1C). Conversely, knockdown of ChREBP did not affect the basal expression of *GLUT*5 but significantly reduced Dex-induced *GLUT*5 expression (Figure 1D). These results suggest that Dex-induced *GLUT*5 expression in Caco-2BBE cells partially depends upon ChREBP.

### Dex induces nuclear translocation of ChREBP in Caco-2BBE cells

**Figure 2.**
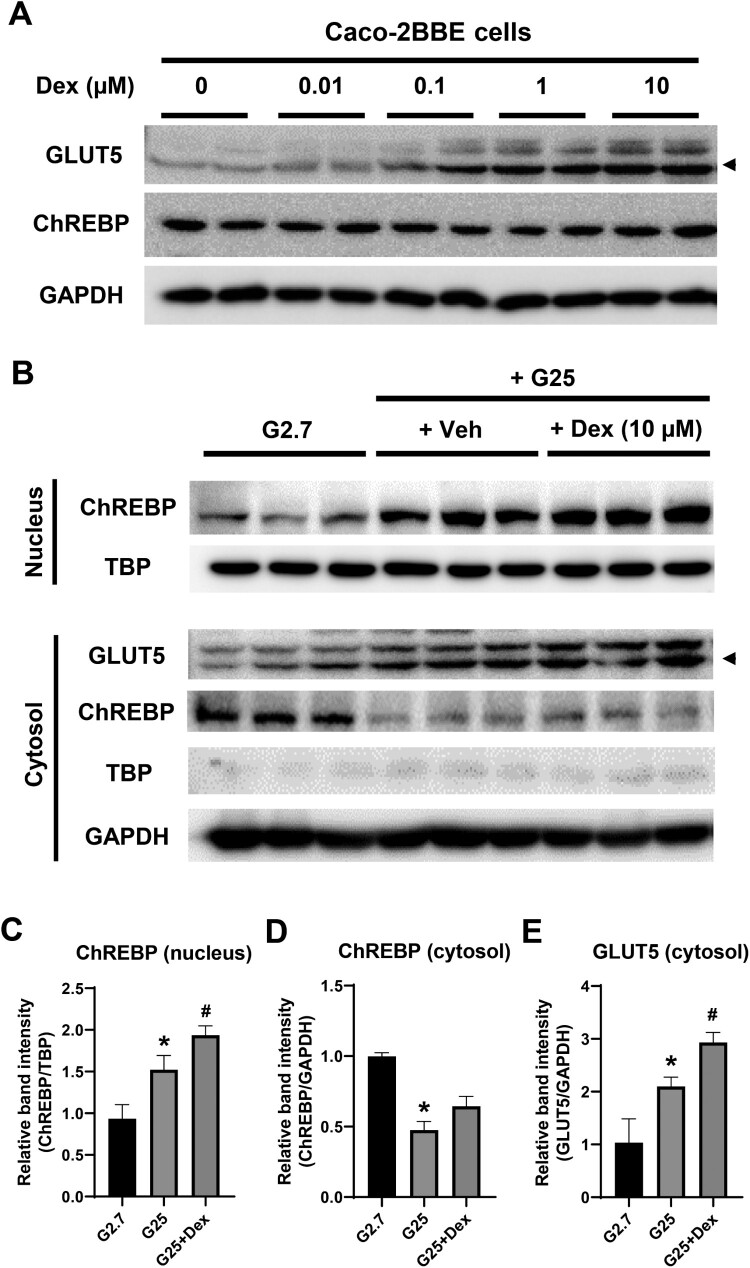
Dexamethasone induces nuclear translocation of ChREBP in Caco-2BBE cells. (A) Caco-2BBE cells were cultured in DMEM containing 25 mM glucose for 24 h after plating. Subsequently, the cells were treated with dexamethasone (Dex) at the indicated doses, followed by western blot analysis after 24 h of Dex treatment. The protein levels of GLUT5 and ChREBP were determined through western blotting, with GAPDH serving as an internal control. (B) Caco-2BBE cells were cultured in DMEM with the indicated glucose levels, followed by 10 μM Dex treatment for 24 h. Following Dex treatment, the levels of nuclear ChREBP or cytoplasmic GLUT5 and ChREBP in the nuclear or cytoplasmic fractions of Caco-2BBE cells were determined using western blotting. TBP was used as an internal control for the nuclear fraction, and GAPDH was used as an internal control for the cytosolic fraction. The protein bands obtained from western blotting were quantified using ImageJ and normalized to TBP or GAPDH for the nuclear or cytoplasmic fractions, respectively. The *arrow head* (◀) indicates the position of GLUT5. *p < 0.05 vs. 2.7 mM glucose-treated group; ^#^*p* < 0.05 vs. 25 mM glucose-treated group.

Although ectopic ChREBP expression increased Dex-induced *GLUT*5 mRNA levels in Caco-2BBE cells, Dex treatment did not affect *ChREBP* mRNA expression. Therefore, we determined whether Dex increased GLUT5 and ChREBP protein levels. Following Dex treatment, GLUT5 protein levels increased dose-dependently, whereas ChREBP protein levels did not increase (Figure 2A). Subsequently, we measured the cellular distribution of ChREBP because of the correlation between its nuclear fraction and activity (Kawaguchi et al. 2001; da Silva Xavier et al. 2006; Dentin et al. 2006) (Figure 2B & C). Under 2.7 mM glucose conditions, ChREBP was abundant in the cytosolic fraction. High glucose concentration (25 mM) increased the translocation of ChREBP into the nucleus, and Dex treatment enhanced this effect. Consistent with these results, GLUT5 levels increased in the presence of 25 mM glucose compared to 2.7 mM glucose and exhibited further increase with Dex treatment. These results suggest that Dex treatment induces nuclear translocation of ChREBP in Caco-2BBE cells.

### Dex increases the activity of the *GLUT5* promoter via ChREBP

**Figure 3.**
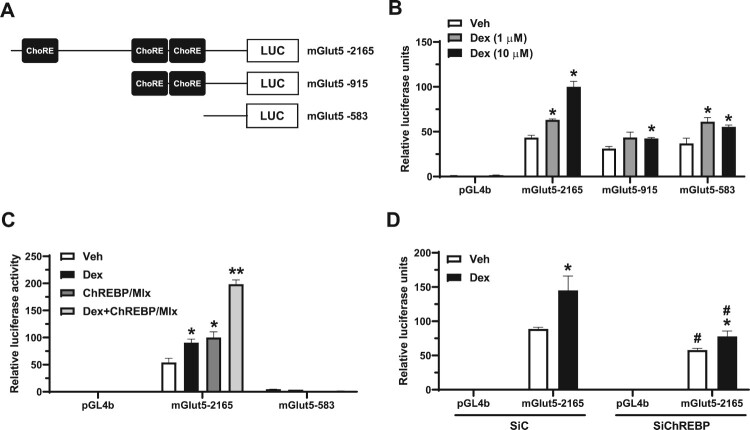
Dexamethasone increases *Glut5* promoter activity, partly through ChoREs. (A) The locations and sequences of carbohydrate response elements (ChoREs) in the mouse *Glut5* (*mGlut5*) promoter, along with a schematic diagram of the deleted *Glut5* promoter-luciferase reporter constructs. (B) Serially deleted *Glut5* promoter-luciferase reporter constructs were transfected into Caco-2BBE cells, with or without Dex (1 or 10 μM). Luciferase activity was measured after 24 h of Dex treatment in Caco-2BBE cells. *p < 0.05. vs. *mGlut5* promoter-luciferase reporter construct-matched, Veh group. (C) Luciferase reporter analysis of the *mGlut5* promoter in the Caco-2BBE cells overexpressing ChREBP/Mlx treated with Dex (10 μM). (D) Luciferase reporter analysis of the *mGlut5* promoter in the Caco-2BBE cells treated with siChREBP (100 pmol) for 48 h. Results were expressed as the fold increase in luciferase activity (mean ± SEM) relative to the control vector pGL4b. *p < 0.05 vs. Veh group; ^#^*p *< 0.05 vs. SiC group.

We investigated whether Dex influences the transcriptional activity of GLUT5 through ChREBP. Previously, we reported that ChREBP increased the mouse *Glut*5 (*mGlut5*) promoter activity by directly binding to the *Glut*5 promoter in Caco-2BBE cells and the mouse intestine [8]. Since ChREBP activates the *Glut5* promoter via ChoRE elements (Oh et al. 2018), we evaluated the putative ChoRE sequences in the *Glut5* promoter of serially deleted promoter constructs (Figure 3A) using Dex treatment. Our results showed that Dex treatment induced the luciferase activity of mGlut5-2165 in Caco-2BBE cells in a dose-dependent manner (Figure 3B). However, the luciferase activity of mGlut5-915 and mGlut5-583 decreased in Caco-2BBE cells treated with Dex compared with the other *Glut5* promoter constructs (Figure 3B). In addition, the Dex-mediated efficacy disappeared in cells transfected with mGlut5-915 or mGlut5-583 (Figure 3B). Additionally, as the upstream ChoRE has been reported to be required for ChREBP/Mlx-mediated *Glut5* promoter activity in the intestine (Oh et al. 2018), the luciferase construct of mGlut5-2165 was selected to test the effect of Dex on the promoter activity of *Glut*5 in the presence or absence of ChREBP/Mlx. Luciferase activity of the mGlut5-2165 construct increased by Dex and was synergistically activated by ChREBP/Mlx (Figure 3C). Enhanced *Glut*5 promoter activity by Dex or ChREBP/Mlx overexpression depended on the ChREBP response elements in the *Glut5* promoter construct (Figure 3C). Consistent with previous results, ChREBP knockdown decreased Dex-induced mGlut5-2165 promoter activity (Figure 3D). Dex-induced *Glut5* promoter activity is mediated by ChREBP-binding elements in the *Glut5* promoter in Caco-2BBE cells.

### Dex-induced intestinal *Glut5* gene expression was blunted in suckling ChREBP-KO mice

**Figure 4.**
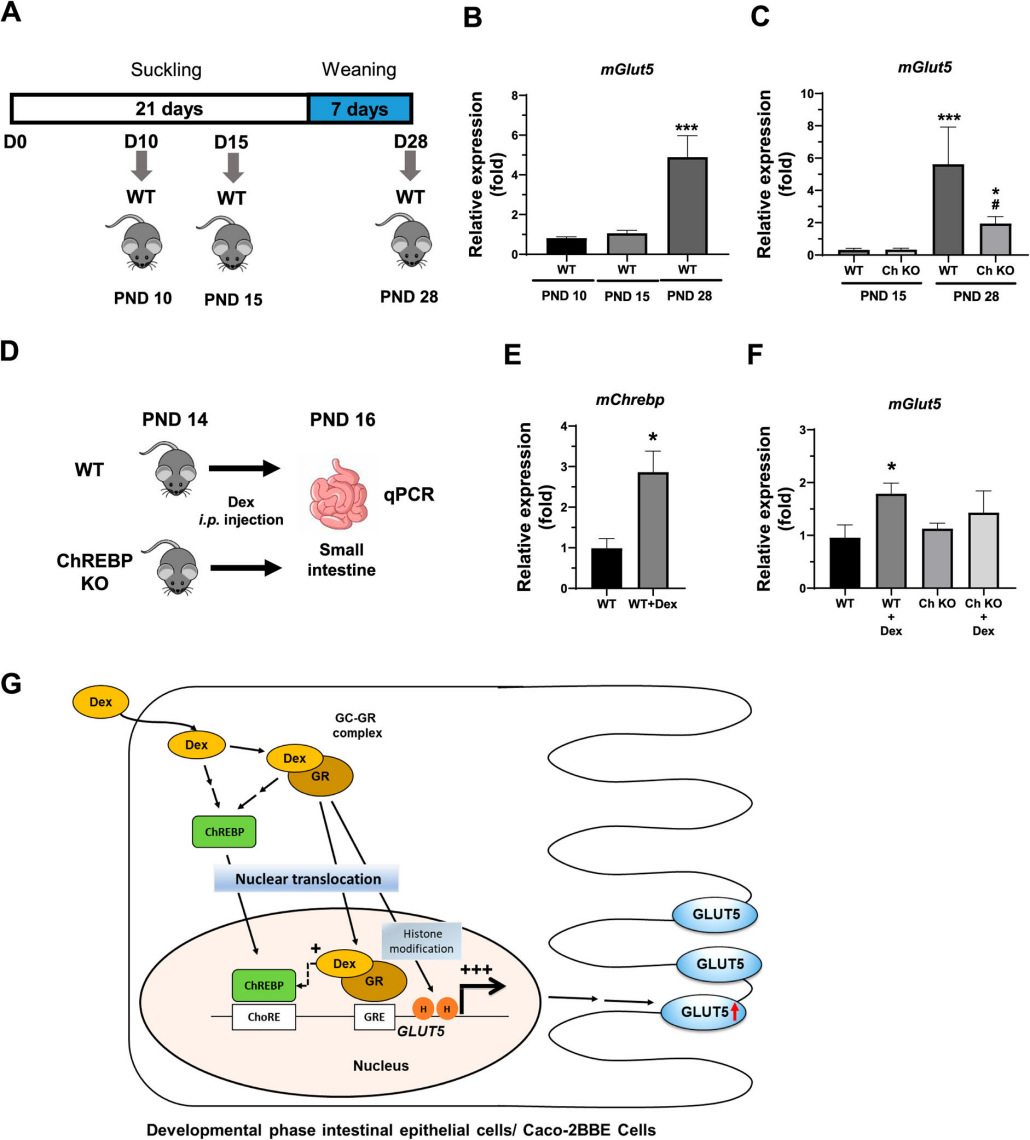
The *Glut5* gene expression is altered in the intestines of mice during the suckling-to-weaning transition. (A) Experimental design for analyzing *Glut5* gene expression during the suckling-to-weaning transition at postnatal day (PND) 10, 15, and 28. (B) qPCR analysis of *Glut5* expression in the duodenum tissues (small intestine) of wild-type (WT) mice at PND 10, 15, and 28. *** *p* < 0.001 vs. PND10 WT group. (C) Analysis of *Glut5* expression in the small intestine of ChREBP-KO mice at PND 15 and 28. ***p < 0.001 vs. PND15 WT group; ^#^*p *< 0.05 vs. PND-matched genotype control. (D) Experimental design for analyzing *Glut5* gene expression in the intestine of Dex-treated mice at PND 14. B6 mice at PND 14 were administered daily intraperitoneal injections of Dex (1 mg/kg) for 3 days, followed by qPCR analysis of duodenum tissues for *Chrebp* (E) and *Glut5* (F). Data are expressed as the mean ± SEM from three independent experiments. *p < 0.05 vs. WT group. (G) The molecular mechanism by which Dex enhances GLUT5 expression during suckling phase and Caco-2BBE cells. Dex treatment facilitates the translocation of ChREBP from the cytoplasm to the nucleus, augmenting GLUT5 expression. Concurrently, Dex binds to the glucocorticoid receptor (GR), and the Dex-GR complex translocates into the nucleus. This complex then activates GLUT5 expression by binding to the glucocorticoid response element (GRE) on the GLUT5 promoter. Additionally, the Dex-GR complex is known to influence histone modifications at the GLUT5 promoter (Mochizuki et al. 2008). The synergistic action of the Dex-ChREBP axis on GLUT5 expression highlights a complex regulatory network governing intestinal GLUT5 expression in response to glucocorticoid signaling.

To determine whether ChREBP mediates the effects of Dex on *GLUT5* expression *in vivo*, *Glut5* expression was measured in the small intestine of Dex-treated WT and ChREBP-KO mice. We first measured *Glut5* expression levels in WT mice on PND 10 (exclusively suckling), PND 15 (maternal milk ingestion > solid food ingestion), and PND 28 (exclusively solid food ingestion) (Figure 4A). While the mRNA levels of *Glut5* were low in the intestine of PND 10 and 15 WT mice, a significant increase in *Glut5* expression was observed in the intestine of PND 28 WT mice (Figure 4B). Although the induction of *Glut5* was observed in the intestine of PND 28 ChREBP-KO mice, it was significantly lower than that in PND 28 WT mice (Figure 4C).

As glucocorticoid signaling was relatively low during the suckling period, pups at PND 14 from WT or ChREBP-KO mice were administered Dex via daily intraperitoneal injection for 3 days (Figure 4D), followed by qPCR analysis for *Glut*5 gene expression. *Glut5* expression exhibited a significant increase in the intestine of WT mice but not in ChREBP-KO mice (Figure 4E–F). These results suggest that ChREBP is required for Dex-induced *Glut*5 gene expression in the small intestine of mice during the suckling period.

## Discussion

The intricate regulation of GLUT5 expression, especially in Dex induction and ChREBP mediation, reveals a multilayered narrative of metabolic regulation that bridges the *in vitro* and *in vivo* paradigms. Our findings underscore the nuanced, dose-dependent amplification of *GLUT5* mRNA in response to Dex treatment without a concomitant alteration in *ChREBP* mRNA levels, highlighting the specificity of glucocorticoid signaling in modulating gene expression in intestinal epithelial cells. While *ChREBP* mRNA levels remained stable, Dex treatment facilitated the nuclear translocation of ChREBP, particularly under elevated glucose conditions (Figure 2), thereby elucidating the potential mechanistic pathways through which glucocorticoids may modulate metabolic gene expression. This spatial regulation of ChREBP, transitioning from the cytosolic to the nuclear fraction, and its correlation with GLUT5 expression, necessitates further exploration into the molecular mechanisms underpinning ChREBP activity and its role in metabolic regulation.

In this study, we focused on the small intestine due to its critical role in fructose absorption, mediated predominantly by GLUT5. The small intestine is a primary organ for absorbing fructose from food, which distinguishes it from other metabolically active organs such as the liver, adipose tissue, and muscle. Although GLUT5 is highly expressed in the small intestine, it is also expressed in the liver, adipose tissue, and muscle, organs for crucial for fructose metabolism (Hajduch et al. 1998; Godoy et al. 2006). In these tissues, fructose metabolism is integral to various physiological processes, including gluconeogenesis in the liver and lipid storage in adipose tissue. Consequently, understanding how ChREBP and Dex influence GLUT5 expression in these contexts could reveal additional layers of metabolic regulation.

The transcriptional activity of ChREBP, known to be regulated by a myriad of factors, including its phosphorylation status, subcellular localization, glucose metabolites, post-translational modification, and protein stability, presents a complex regulatory network (Ortega-Prieto and Postic 2019). ChREBP is typically maintained in the cytoplasm in a phosphorylated, inactive state through the action of AMPK (Kawaguchi et al. 2002). The activation and nuclear translocation of ChREBP require dephosphorylation by protein phosphatase 2A (PP2A) in the liver (Kawaguchi et al. 2001). Although there is no direct evidence linking PP2A to Dex-mediated signaling pathways, our findings of Dex-induced ChREBP nuclear translocation in Caco-2BBE cells suggest a potential, yet unexplored, role for PP2A in mediating this response. This postulation is supported by the broader understanding of PP2A as a modulator of transcription factor activity, hinting at its capacity to serve as a nexus for Dex signaling and ChREBP activation. Given these premises, future investigative efforts are warranted to dissect the relationship between Dex signaling and PP2A activity. This should include a focused inquiry into the role of PP2A in the dephosphorylation and nuclear mobilization of ChREBP, which could illuminate novel regulatory mechanisms pertinent to glucocorticoid-induced metabolic gene expression.

The *in vivo* findings, particularly the attenuated *Glut5* expression in ChREBP-KO mice upon Dex treatment (Figure 4), corroborate the *in vitro* findings and underscore the physiological relevance of ChREBP in mediating glucocorticoid-induced metabolic gene expression. The developmental course of *Glut5* expression, transitioning from exclusive suckling to solid food ingestion, and its modulation by ChREBP, open avenues for exploring the developmental and metabolic implications of altered *Glut5* expression during different life stages.

Moreover, the intersection of glucocorticoid signaling and ChREBP in mediating *Glut5* expression during critical developmental transitions, such as suckling and weaning, has not been thoroughly elucidated. Although ChREBP has been identified as an essential transcription factor that maintains *Glut5* expression in the intestinal tissues of mice (Oh et al. 2018), the molecular mechanism underlying glucocorticoid signaling-induced *Glut5* expression, potentially mediated by epigenetic alterations, remains to be explored in detail. The potential facilitation of ChREBP’s access to the *Glut5* promoter in Caco-2BBE cells by Dex, possibly through the methylation or acetylation of chromatin, presents an intriguing avenue for further research.

Considering these findings, our study provides a foundational understanding of the role of ChREBP in Dex-induced *Glut5* gene expression (Figure 4G). It reveals that Dex enhances *Glut5* expression in Caco-2BBE cells via ChREBP, suggesting a complex interplay between glucocorticoid signaling, ChREBP activity, and GLUT5 expression. Future investigations may delve deeper into the molecular mechanisms underlying ChREBP-mediated regulation of GLUT5 and explore potential co-regulators, as well as the downstream metabolic consequences of altered GLUT5 expression. Furthermore, exploring the physiological and metabolic implications of modulated GLUT5 expression within various dietary and developmental contexts may provide insights into the roles of glucocorticoids and ChREBP signaling in metabolic diseases and disorders.

## Conclusions

This study elucidates the pivotal role of ChREBP in mediating Dex-induced *Glut5* expression in intestinal epithelial cells, revealing that Dex treatment not only enhances *Glut5* expression but also promotes the nuclear translocation of ChREBP. Dex-induced *Glut5* promoter activity relies on the binding of ChREBP to the ChoRE element in the *Glut5* gene, providing novel insights into glucocorticoid-induced *Glut5* regulation in the immature intestine and paving the way for further research into understanding the complex molecular signaling between post-translational modifications of ChREBP and epigenetic changes in *Glut5*.
